# Association of spironolactone use with risk of urinary tract cancer in the general population: A matched population-based cohort study

**DOI:** 10.1371/journal.pone.0300391

**Published:** 2024-03-27

**Authors:** Liang-Cheng Chen, Hsuan-Ju Yang, Ben-Hui Yu, Moon-Sing Lee, Hon-Yi Lin, Wen-Yen Chiou, Dai-Wei Liu, Feng-Chun Hsu, Chia-Hui Chew, Shih-Kai Hung

**Affiliations:** 1 Department of Radiation Oncology, Dalin Tzu Chi Hospital, Buddhist Tzu Chi Medical Foundation, Chiayi, Taiwan; 2 School of Medicine, Tzu Chi University, Hualien, Taiwan; 3 Department of Computer Science and Information Engineering, National Cheng Kung University, Tainan City, Taiwan; 4 Department of Biomedical Sciences, National Chung Cheng University, Min-Hsiung, Chia-Yi, Taiwan; 5 Department of Radiation Oncology, Hualien Tzu Chi Hospital, Buddhist Tzu Chi Medical Foundation, Hualien City, Taiwan; University of Luebeck, GERMANY

## Abstract

**Purpose:**

The correlation between spironolactone usage and cancer risk has sparked interest. The objective of this study is to examine the association between spironolactone use and the incidence of urinary tract cancer in the general population.

**Methods:**

We conducted a matched population-based cohort study. The study population was obtained from the Taiwan National Health Insurance Research Database (TNHIRD) during the period from 2000 to 2016. The multivariate Cox proportional hazard model was performed to examine the impact of spironolactone use on the risk of urinary tract cancer. A total of 8,608 individuals exposed to spironolactone were exact matched by 1:1 ratio with unexposed controls on factors including age, gender, comorbidities, CCI scores and socioeconomic status. The incidences of urinary tract cancer, including prostate, renal and bladder cancer, were estimated in both spironolactone exposed and non-exposed cohorts.

**Results:**

After adjusting for confounding variables, the multivariate Cox regression analysis showed no significant association between spironolactone exposure and urinary tract cancer incidence, including bladder (adjusted hazard ratio [aHR] = 1.19, 95% confidence interval [CI] = 0.72–1.96, p = 0.50), renal (aHR = 1.75, 95% CI = 0.99–3.07, p = 0.053), and prostate cancer (aHR = 0.67, 95% CI = 0.43–1.04, p = 0.07). When the population was stratified into low (cumulative dose < = 29,300 mg) and high (cumulative dose >29,300 mg) dose of spironolactone, only high dose of spironolactone use was significantly associated with a reduced risk of prostate cancer (aHR = 0.45, 95% CI = 0.23–0.89, p = 0.02), while being associated with an elevated risk of renal cancer (aHR = 2.09, 95% CI = 1.07–4.08, p = 0.03). However, no clear cumulative dose-response relationship was observed in theses associations.

**Conclusions:**

High cumulative dose of spironolactone may be potentially associated with a decreased incidence of prostate cancer and an increased incidence of renal cancer, while no significant association was observed with bladder cancer incidence. However, given the lack of support from the dose-response pattern, the available evidence is inconclusive to establish a definitive association between spironolactone use and urinary tract cancer.

## Introduction

Spironolactone is a frequently prescribed medication for treating fluid accumulation resulting from heart failure [1] and ascites due to liver cirrhosis [2]. The use of spironolactone contributed to a significant reduction in the risk of all-cause mortality by 30% and the frequency of hospitalization for worsening heart failure by 35% [1]. However, with the increasing usage of spironolactone in clinical settings, concerns regarding its potential association with cancer risk have arisen.

Long-term exposure to medication may increase or decrease the risk of site-specific cancers [3–5]. Prostate cancer is regarded as androgen-sensitive tumor [6] and the spironolactone use was indicated to be associated with risk of prostate cancer by means of anti-androgenic effects [6, 7]. Additionally, spironolactone is also an aldosterone antagonist [8] and reduced incidence of prostate cancer among men with heart failure [9] and lower risk of bladder cancer in female with hypertension [7] were observed in spironolactone users. Nevertheless, the link between the spironolactone use and the risk of developing urinary tract cancer has not been established, as previous studies have yielded inconsistent results [7, 10, 11].

Potential drug effects on cancer incidence can be efficiently explored by analyzing large-scale healthcare data [5]. Different to the majority of previous studies, we utilized an exact matched cohort study design to investigate the relationship between spironolactone use and the risk of urinary tract cancer, and the dose-response pattern was examined to reveal levels of hazard ratio and dosage for spironolactone to individuals were exposed.

## Methods

This study adopted a population-based cohort design and employed the method of exact match to control for potential confounding variables. Exact match is considered to be a rigorous method of controlling confounding factors because it ensures that the groups being compared are identical in terms of the matched variables. In this study, five variables, including age, gender, comorbidities, Charlson Comorbidity Index (CCI) scores, and socioeconomic status (insured classification), were identified as potential confounding factors (as shown in Table 1). To mitigate the influence of these confounding factors, subjects from the spironolactone-exposed and non-exposed groups were precisely matched with 1:1 ratio based on these variables, ensuring that both groups shared identical values for these potential confounders.

**Table 1:** Demographics and clinical information of study population.

	Spironolactone		
	Exposed N = 8,606 (%)	Non-exposed N = 8,606 (%)	p-value
^a^Age group (years)			1.00
20–60	3815 (44.3)		
60–70	1893 (21.9)		
70–80	2120 (24.6)		
80–100	780 (9.0)		
^a^Gender			1.00
Male	4517 (52.4)		
Female	4091 (47.5)		
^a^Comorbidities			1.00
COPD	1451 (16.8)		
Hypertension	4680 (54.3)		
Heart failure	1524 (17.7)		
DM	2406 (27.9)		
Liver disease	2398 (27.8)		
Abdominal pain	883 (10.2)		
Liver cirrhosis	1254 (14.5)		
CKD	214 (2.4)		
^a^Insured classification			1.00
Class I	2422 (28.1)		
Class II	79 (0.9)		
Class III	3449 (40.0)		
Class IV	2658 (30.8)		
^a^CCI score			1.00
0	1530 (17.7)		
1	2074 (24.0)		
2	1650 (19.1)		
3	1185 (13.7)		
> = 4	2169 (25.2)		
Other drug use			
ACEI	272 (3.1)	92 (1.0)	<0.001
Digoxin	1513 (17.5)	362 (4.2)	<0.001
Metformin	237 (2.7)	236 (2.7)	0.96
Glucocorticoids	4839 (56.2)	4078 (47.3)	<0.001
Methoxypsoralens	0	0	--
NSAID-aspirin	3037 (35.2)	2161 (25.1)	<0.001
NSAID-nonaspirin	6437 (74.7)	6076 (70.5)	<0.001
Statins	1327 (15.4)	1190 (13.8)	<0.01
Geographic region			<0.001
North	3237 (37.6)	4225 (49.0)	
Central	2882 (33.4)	1837 (21.3)	
East	263 (3.0)	242 (2.8)	
South	2226 (25.8)	2304 (26.7)	
Urbanization level			<0.001
Metropolis	1710 (19.8)	1990 (23.1)	
Satellite cities	4019 (46.6)	3950 (45.8)	
Rural areas	2879 (33.4)	2668 (30.9)	

COPD, chronic obstructive pulmonary disease; DM, diabetes mellitus; CKD, chronic kidney disease; ACEI, angiotensin converting enzyme inhibitors; NSAID, non-steroidal anti-inflammatory drugs

^a^ These variables were used for a 1:1 exact matching between the spironolactone-exposed and non-exposed cohorts.

### Database

The study population was obtained from Taiwan National Health Insurance Research Database (TNHIRD) during the period from 2000 to 2016. The TNHIRD contains comprehensive information concerning the records of diagnosis and clinical treatment of approximately 99% of people from inpatient, outpatient, and emergency departments [12] and are evaluated strictly by the National Health Insurance Administration (NHIA) [13]. The National Health Insurance Administration performs quarterly expert reviews on claims filed by each medical institution, and the information obtained from the TNHIRD is considered both complete and accurate [13]. The release of the drug use and clinical information in this research database was approved for research purposes by the Health and Welfare Data Science Center (HWDC), Ministry of Health and Welfare, Taiwan. The confidentiality assurances were addressed by following the data regulations of the HWDC.

This study was performed with the approval of Institutional Review Board (IRB) of Dalin Tzu Chi Hospital of Buddhist Tzu Chi Medical Foundation (approval number, B10704014). Data were accessed and extracted from TNHIRD on 01 February 2019, and the information of individual participants cannot be identified by researchers either during or after data collection. Informed consent was not required as the dataset is de-identified.

### Study subjects

**Fig 1:**
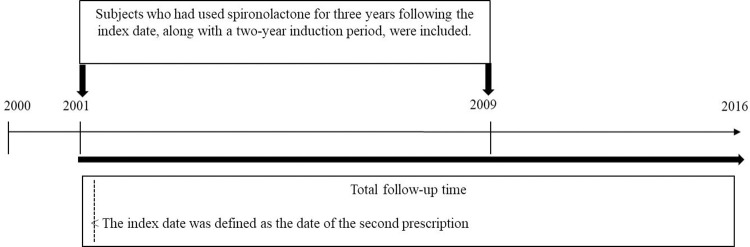
The demonstration of study population selection process.

The study population selection process is illustrated in **Fig 1**. Subjects who had at least two prescriptions for spironolactone from 2000 to 2016 were identified. The date of the second prescription for spironolactone was defined as the index date for each subject, rather than the date of the first prescription.

During the period of 2001 and 2009, the subjects included in the spironolactone-exposed cohort were those who had received a cumulative dosage for three years following the index date, along with a two-year induction period. This period of five years was considered reasonable for assessing the effect of the drug on the risk of cancer incidence, as cancers often have substantial periods to develop [16]. In order to reduce the potential impact of noncompliance on our findings, patients with fewer than 40 prescriptions were excluded. In addition, those who died or developed cancer within 5 years of the index date were also excluded. Therefore, between the period of 2001 and 2009, a total of 10,600 patients aged 20 to 100 years old were identified and 8,608 spironolactone-exposed subjects were included and exact matched 1:1 with unexposed controls based on age, gender, comorbidities, CCI scores, and socioeconomic status. The related characteristics and clinical information were summarized in **Table 1**.

### Study outcomes

We defined outcomes as the first incidence of cancer during the follow-up period, with cancers identified based on the International Classification of Disease, Ninth Revision, Clinical Modification (ICD-9-CM codes). The urinary tract cancer comprised three distinct cancer types, including bladder cancer (ICD-9-CM 188), renal cancer (ICD-9-CM 1890 and 1891) and prostate cancer (ICD-9-CM 185). The incidence rates of these three cancer types were measured in both spironolactone exposed and non-exposed cohorts.

### Other characteristics

We included variables which were considered as potential confounders in this study, such as (i) comorbidities, including chronic obstructive pulmonary disease (COPD), diabetes mellitus (DM), chronic kidney disease (CKD), liver cirrhosis, hypertension, heart failure, liver disease, and abdominal pain; (ii) Charlson Comorbidity Index (CCI) scores derived from 19 different medical condition categories [16]; and (iii) other medications, such as angiotensin-converting enzyme (ACE) inhibitors, digoxin, metformin, glucocorticoids, methoxypsoralens, non-steroidal anti-inflammatory drugs (NSAID-aspirin and NSAID-nonaspirin), and statins. These comorbidities and medication use were considered as covariates in our data analysis. In addition, demographic variables such as age, gender, geographic region, urbanization level, and socioeconomic status (based on family income) were included in our statistical analysis to reduce bias resulting from lifestyle factors.

### Statistical analysis

The basic characteristics of the spironolactone-exposed and non-exposed cohorts were compared using the Chi-square test. A multivariate Cox proportional hazard model was used to calculate the adjusted hazard ratio (aHR) and 95% confidence interval (CI) to examine the incidence of cancer in the spironolactone-exposed cohort compared to the matched non-exposed cohort. In addition, associations identified between different levels of spironolactone use and cancer risks were further assessed using cumulative dose-response pattern analysis. We conducted further examination of a cumulative dose-response pattern for the spironolactone-exposed cohort, and illustrated the relationship between the cumulative dose of spironolactone and the aHR of cancer risks through the cumulative dose-response pattern.

All statistical analyses were conducted using SAS software (version 9.2; SAS Institute, Inc., Cary, NC). A two-sided P-value of less than 0.05 was considered statistically significant.

## Results

After adjusting for covariates (see all variables in **Table 1**), the multivariate Cox regression analysis showed no significant association between spironolactone exposure and urinary tract cancer incidence, including bladder (aHR = 1.19, 95% confidence interval [CI] = 0.72–1.96, p = 0.50), renal (aHR = 1.75, 95% CI = 0.99–3.07, p = 0.053), and prostate cancer (aHR = 0.67, 95% CI = 0.43–1.04, p = 0.07).

**Fig 2:**
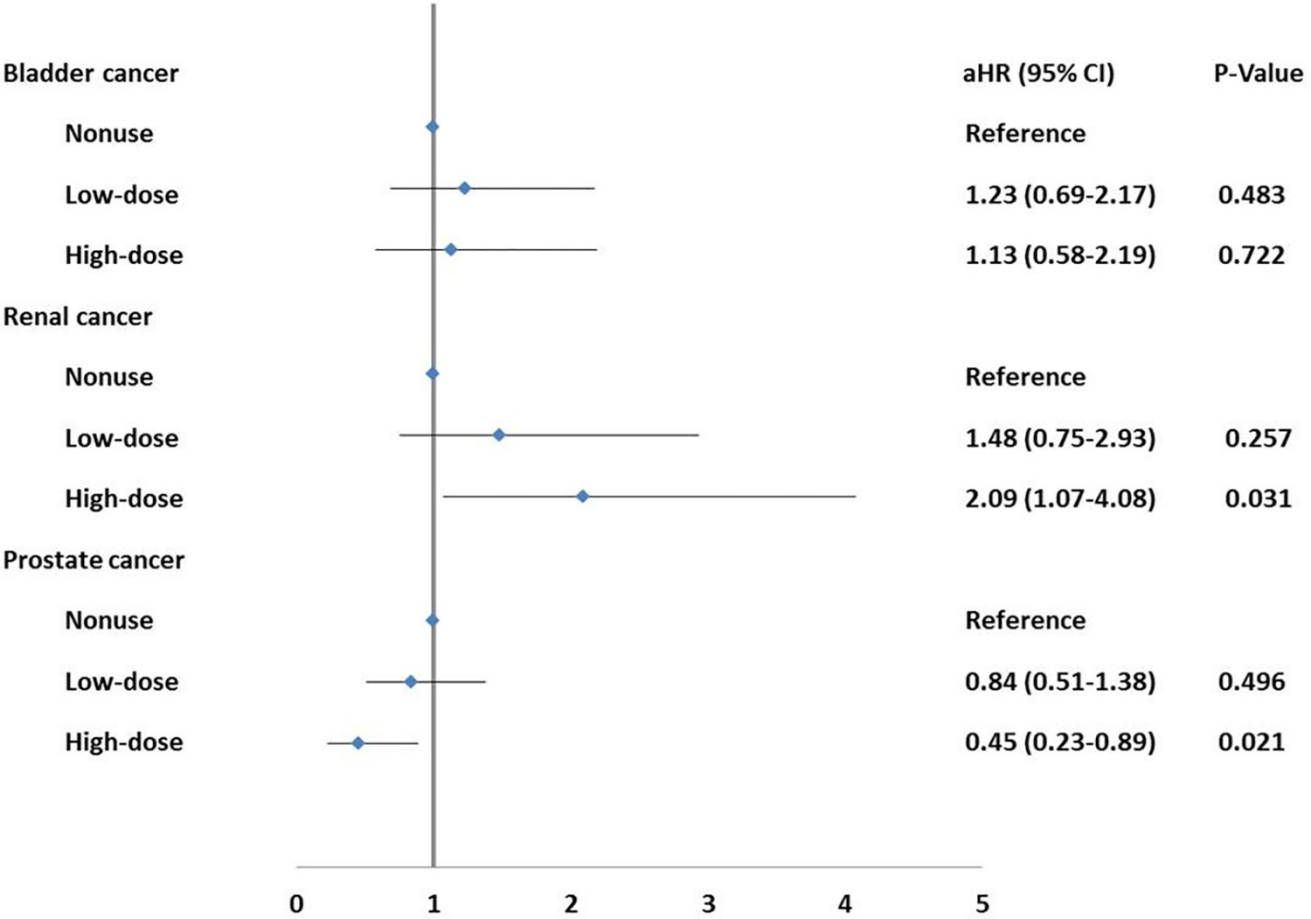
The association between spironolactone exposure and risk of upper urinary tract cancers among different groups of spironolactone use.

We further examined the association between varying cumulative doses of spironolactone and urinary tract cancer risks. The cumulative dose of 29,300 mg was identified to evenly divide the total number of subjects into two groups. Consequently, this cut-off of 29,300mg was utilized to classify the utilization of spironolactone into high and low-dose categories through a 1:1 exact match, resulting in 4,466 subjects with a cumulative dose lower than 29,300 mg and 4,142 subjects with a cumulative dose higher than 29,300 mg, respectively. When the spironolactone users was stratified into low-dose (cumulative dose < = 29,300 mg) and high-dose (cumulative dose >29,300 mg) groups, patients in the high-dose group had a significantly increased risk of renal cancer (aHR = 2.09, 95% CI = 1.07–4.08, p = 0.03) compared to those without spironolactone exposure. In contrast, a significant reduction in the risk of prostate cancer was observed in the high-dose group (aHR = 0.45, 95% CI = 0.23–0.89, p = 0.02) (see **Fig 2**). However, no significant association between spironolactone dosage and the risk of bladder cancer was found.

**Fig 3:**
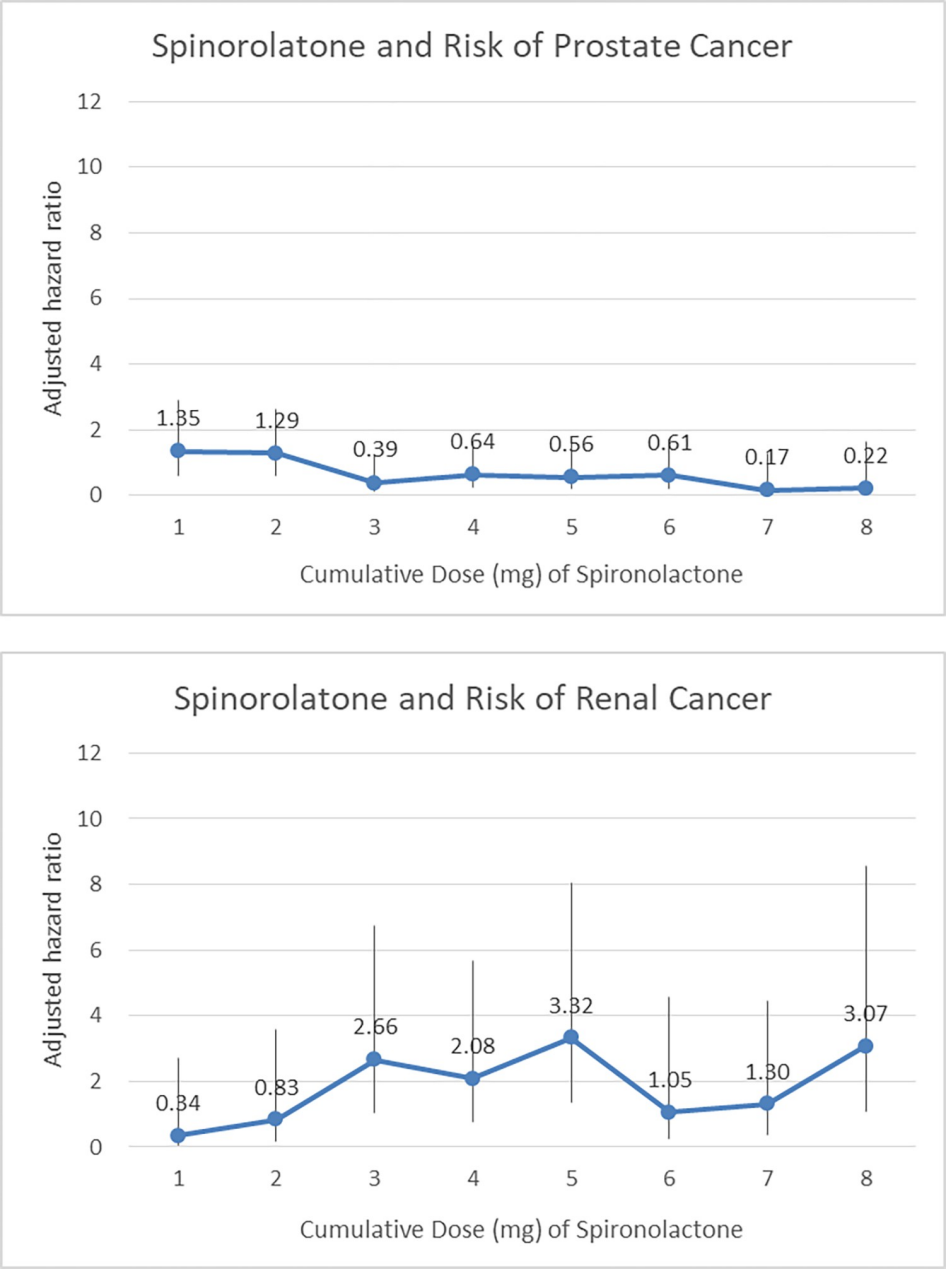
The adjusted hazard ratios of prostate and renal cancers at different levels of cumulative dose of spironolactone.

For a more in-depth analysis of the dose-response pattern, we examined whether there was a significant associated trend between the cumulative dose of spironolactone and the aHR of cancer risk. Our analysis revealed a slightly progressive decreasing in aHR for prostate cancer with increasing cumulative doses of spironolactone, whereas not reaching statistically significance (F-value, 1.92; p = 0.22). Similarly, no such trend was observed for renal cancer (F-value, 0.94; p = 0.37; see **Fig 3**). Taken together, the trend of consistent change of spironolactone for relating to urinary tract cancer was not evident since the significance of the dose-response pattern was not observed.

We also observed the risks of prostate and renal cancer in terms of cumulative dose and duration of exposure to spironolactone. As shown in **Table 2**, the aHR for prostate cancer gradually decreased from 1.32 (p = 0.338) for those in the lowest 25% of cumulative spironolactone dose to 0.20 (p = 0.024) for those in the highest 25% of cumulative spironolactone dose. Similar results were observed for the change in aHR in terms of exposure duration of spironolactone. The aHR for prostate cancer gradually decreased from 1.15 for those with less than 862 days of spironolactone use to 0.55 for those with more than 1148 days of spironolactone use. For renal cancer risk, the aHR for renal cancer gradually increased from 0.57 for those in the lowest 25% of cumulative spironolactone dose to 2.04 for those in the highest 25% of cumulative spironolactone dose. In addition, there was an incremental increase in renal cancer risk with longer exposure time to spironolactone. The aHR for renal cancer gradually increased from 0.83 (p = 0.748) for those with less than 862 days of spironolactone use to 2.30 (p = 0.033) for those with more than 1148 days of spironolactone use (see **Table 2**).

**Table 2:** Association between the intensity of spironolactone use and the risk of renal and prostate cancer.

	Prostate cancer			Renal cancer		
	aHR	95% CI	p value	aHR	95% CI	p value
Spironolactone exposure						
No	Reference			Reference		
Yes	0.67	0.43–1.04	0.074	1.75	0.99–3.07	0.053
Cumulative dose						
None	Reference			Reference		
Lowest quartile	1.32	0.75–2.30	0.338	0.57	0.16–1.96	0.370
Quartile 2	0.52	0.24–1.11	0.090	2.37	1.12–5.01	0.024
Quartile3	0.59	0.28–1.25	0.169	2.24	1.01–5.01	0.048
Highest quartile	0.20	0.05–0.81	0.024	2.04	0.89–4.69	0.094
Exposure duration (days)						
None	Reference			Reference		
< = 862	1.15	0.60–2.22	0.671	0.83	0.27–2.54	0.748
863–1092	0.25	0.08–0.79	0.019	1.73	0.73–4.13	0.215
1093–1147	0.77	0.40–1.47	0.428	2.15	0.99–4.66	0.052
> = 1148	0.55	0.25–1.17	0.119	2.30	1.07–4.96	0.033

Abbreviation: aHR, adjusted hazard ratio; 95% CI, 95% confident interval

Note: aHR was obtained from multivariate Cox regression analysis by adjusting all variables listed on Table 1, including age, gender, comorbidities, insured classification (socioeconomic status), Charlson comorbidity index, other drug uses, geographic region, and urbanization level

Overall, whether the change in aHR assessed in terms of cumulative dose or duration of exposure to spironolactone, we observed a decreasing trend in prostate cancer risk and an increasing trend in renal cancer risk. However, there was no similar result observed for bladder cancer.

### Discussion

This study utilized a methodology of exact matching to establish a cohort study design, with the objective of investigating the correlation between spironolactone usage and risk of urinary tract cancer in the general population. After controlling for the confounding effects of medication and baseline characteristics by exact match method, our findings revealed that only high cumulative dose of spironolactone use was significantly associated with a reduced risk of prostate cancer, while being associated with an elevated risk of renal cancer. Nonetheless, the absence of a cumulative dose-response pattern precludes the identification of a consistent trend in this relationship.

Our study deviates from previous research [7, 11, 14] as we did not observe a significant decrease in the risk of prostate cancer among men prescribed spironolactone (see **Table 2**), while a noteworthy reduction in the risk of prostate cancer was observed only in those receiving high cumulative doses of spironolactone (see **Fig 2**). Men with high cumulative spironolactone exposure demonstrated a 55% lower risk of prostate cancer compared to those who did not take this medication, implying the potential requirement for a certain threshold of exposure to influence cancer risk. Spironolactone is hypothesized to be associated with reduced risk of prostate cancer due to its dual properties as an anti-androgen and blocker of aldosterone receptors in the renal tubules [14–16]. Beckmann et al. [14] reported a 17% reduction in the risk of prostate cancer among men exposed to spironolactone (odds ratio, 0.83; 95% CI, 0.76–0.89). Nevertheless, it is crucial to interpret the relationship between spironolactone use and cancer risk with caution as the certainty of the evidence was assessed as very low [10]. Furthermore, our study also did not observe a cumulative dose-response pattern. The available evidence concerning the association between the utilization of spironolactone and the risk of developing prostate cancer lacks sufficient adequacy.

Similarly, a significant increased risk of renal cancer was only found in those with high doses of spironolactone exposure, with the risk being approximately two-fold higher than in individuals not exposed to spironolactone. However, the absence of a dose-response pattern undermines the plausibility of a relationship between the drug and cancer. In the limited previous studies, no significant association between spironolactone use and renal cancer incidence has been reported [10, 11]. Additionally, while a previous experimental study reported that spironolactone reduced proliferation of renal cancer cells by blocking the mineralocorticoid receptor and affecting K-RAS expression [17], this finding contradicts results from prior clinical observations [7, 10, 11] as well as our study. Therefore, caution must be exercised in extrapolating the inhibitory effect of spironolactone treatment on aldosterone-dependent growth of renal cell carcinoma to the general population.

In this study, we implemented rigorous control measures to account for confounding factors, including age, gender, comorbidities, and socioeconomic status, in order to address the question of the association between spironolactone and urinary tract cancer risk. Based on our comprehensive research, we did not find conclusive evidence to support the notion that spironolactone use has a significant impact on the risk of urinary tract cancers. Indeed, a systematic literature review of randomized controlled trials yielded no qualitative or quantitative evidence supporting the association between commonly used antihypertensive drugs and cancer development [18], suggesting that these medications may not be associated with urinary tract cancer risk. Moreover, although experimental data indicated that spironolactone may exhibit antitumor effects by influencing homology-directed repair in cancer cells [19], it remains questionable whether these effects can translate into clinically meaningful outcomes in humans. Therefore, further investigation is warranted to elucidate the potential role of spironolactone in the prevention or development of urinary tract cancers.

### Strengths and limitations

The strength of this study lies in its utilization of an exact match method to control potential variances between two cohorts, ensuring that any differences in outcomes can be confidently attributed to the intervention. Previous studies have rarely reported divergent outcomes regarding the association between spironolactone and urinary tract cancer risk. Our well-controlled observational study provides an additional perspective, raising the possibility that the observed association may be attributable to chance which is similar to recent research on the incidence of cancer associated with statin use [20].

This study has certain limitations. Firstly, due to the fact that cancer may take many years or even decades to manifest, relying solely on our results to draw conclusive evidence may be inadequate. Secondly, the absence of certain information, such as family history of cancer, may have influenced the outcome of our study and should be taken into account in future research. Thirdly, we have to acknowledge that the occurrence of cancer is a complex process influenced by various factors, including environmental exposure, lifestyle, viral infections, and imbalances in hormones and the immune system. However, these factors could not be included in our analysis due to challenges in obtaining relevant data. Finally, controlling for the variance resulting from noncompliance with spironolactone usage is challenging. However, in our study, we implemented an exclusion criterion for individuals with fewer than 40 prescriptions for spironolactone. This exclusionary approach may have mitigated the potential impact of noncompliance on our findings, reducing its influence to a negligible extent.

### Conclusion

In conclusion, a high cumulative dose of spironolactone may be potentially associated with a decreased incidence of prostate cancer and an increased incidence of renal cancer, while no significant association was observed with bladder cancer incidence. However, given the lack of support from the dose-response pattern, the available evidence is inconclusive to establish a definitive association between spironolactone use and urinary tract cancers.
